# Using Linked Data for Mining Drug-Drug Interactions in Electronic Health Records

**Published:** 2013

**Authors:** Jyotishman Pathak, Richard C. Kiefer, Christopher G. Chute

**Affiliations:** Department of Health Sciences Research, Mayo Clinic, Rochester, MN, USA

**Keywords:** Electronic health records, Drug-drug interactions, Semantic Web, Federated querying

## Abstract

By nature, healthcare data is highly complex and voluminous. While on one hand, it provides unprecedented opportunities to identify hidden and unknown relationships between patients and treatment outcomes, or drugs and allergic reactions for given individuals, representing and querying large network datasets poses significant technical challenges. In this research, we study the use of Semantic Web and Linked Data technologies for identifying drug-drug interaction (DDI) information from publicly available resources, and determining if such interactions were observed using real patient data. Specifically, we apply Linked Data principles and technologies for representing patient data from electronic health records (EHRs) at Mayo Clinic as Resource Description Framework (RDF), and identify potential drug-drug interactions (PDDIs) for widely prescribed cardiovascular and gastroenterology drugs. Our results from the proof-of-concept study demonstrate the potential of applying such a methodology to study patient health outcomes as well as enabling genome-guided drug therapies and treatment interventions.

## Introduction

An important aspect in realizing the vision of translational research lies in the ability to access, integrate, analyze and manage multiple and heterogeneous datasets within and across functional domains. This necessitates a systematic study of clinical phenotypes and health-related treatment outcomes to better understand the impact of effective patient-care management. The Semantic Web, and its related tools and technologies provide the underlying infrastructure for large-scale data integration and knowledge acquisition, and are being increasingly adopted by the biological, clinical and translational science communities to serve their information management and querying requirements. In particular, the Linked Open Data (LOD [1]) community project at the World Wide Web Consortium (W3C) is publishing various open data sets as Resource Description Framework (RDF [2]) on the Web and extending it by setting RDF links between data items from different data sources containing information about genes, proteins, pathways, diseases, and drugs. While this presents a very powerful platform for federated querying and heterogeneous data integration, its true potential can only be realized when combining such information with “real” patient data from electronic health records. However, in practice, due to several privacy, security, ethical, policy and confidentiality issues, patient data is closely guarded and monitored for unauthorized access within institutional firewall boundaries. Consequently, projects such as the LOD rely on “fake” and “curated” patient data that do not represent the inherent idiosyncrasies and complexities of information contained within an electronic health record system.

In this manuscript, we report early experiences by leveraging our prior work [3, 4] in applying linked data principles for representing patient data from electronic health record systems at Mayo Clinic to study drug-drug interaction patterns for cardiovascular and gastroenterology drugs. In particular, we use open-source tooling and standardized ontologies for creating materialized RDF graphs from Mayo’s clinical enterprise warehouse and demonstrate federated querying for potential drug-drug interaction (DDI) information using public data available from the Linked Open Data cloud. It is well-known that adverse drug events are a major health risk, and DDIs are one of the causes of such events. However, while thousands of DDIs have been reported, only a handful are worth any attention. Furthermore, a set of DDIs that suit one medical center or patient care facility might not be entirely appropriate to others. Consequently, there is significant research and on-going debate on how DDI information can be leveraged for better care, particularly in the context of clinical decision support and EHRs [5].

The work presented in this study is informed by such advances and focuses primarily on mining DDI information from EHR systems using Semantic Web technologies. Specifically, our proof-of-concept described in this manuscript is based on DDI information for Clopidogrel (Plavix), Warfarin (Coumadin), and multiple Protein Pump Inhibitors. Our reasoning behind selecting these drugs for demonstration of our methods was not only due to the fact that these are commonly prescribed medications, but also because existing literature [6, 7] have demonstrated the role of genes to guide the drug administration and dosing recommendations—an area of future interest and relevance to our project [4]. The preliminary results from our study further illustrate the strong potential for considering Semantic Web technologies to enabling Web-scale data integration and federation in biomedical research and development.

In what follows, we provide a brief background to Mayo Clinic’s EHR and datawarehousing systems as well as emerging Semantic Web technologies and their benefits.

## Background and Materials

### Enterprise Clinical Data Warehouse at Mayo Clinic

Mayo Clinic has a history of over 100 years in organizing patient records to support research and quality improvements [8]. Starting with structured paper documents storing patient information about laboratory results or physical examination findings, Mayo has supported the notion of explicitly missing information since 1907. Around 1990, efforts to integrate and organize information into semantically well-formed structure were initiated in tandem with infrastructure development for registry creation and information retrieval. The Mayo Clinic Life Sciences System (MCLSS [9]) is a rich clinical data repository maintained by the Enterprise Data Warehousing Section of the Department of Information Technology. MCLSS contains patient demographics, diagnoses, hospital, laboratory, flowsheet, clinical notes and pathology data obtained from multiple clinical and hospital source systems within Mayo Clinic at Rochester, Minnesota. Data in MCLSS is accessed via the Data Discovery and Query Builder (DDQB) toolset, consisting of a web-based GUI application and programmatic API. Investigators, study staff and data retrieval specialists can utilize DDQB and MCLSS to rapidly and efficiently search millions of patient records. Users are able to quickly build, save and share complex queries without programming or database knowledge. A unique text search engine provides the capability to rapidly search for specific words and phrases within unstructured text documents, such as clinical notes and pathology reports, freeing investigators from many hours of tedious manual chart reviews. Data found by DDQB can be exported into CSV, TAB or Excel files for portability. It implements full data authorization and audit logging to ensure data security standards are met.

For this project, we leverage MCLSS to access and retrieve patient demographic, diagnoses and medication data, and represent such knowledge using Resource Description Framework (RDF). We discuss additional details and processes involved in the remainder of this manuscript.

### Semantic Web and Related Technologies

A key benefit of using Semantic Web technologies is that it is a rigorous mechanism of defining and linking data using Web protocols in a way, such that, the data can be used by machines not just for display, but for automation, integration, and reuse of across various applications. Specifically, an “attractive” element of the Semantic Web is its simple data model—RDF—that represents data as a labeled graph connecting resources and their property values with labeled edges representing properties. The graph can be structurally parsed into a set of triples (subject, predicate, object), making it very general and easy to express any type of data. Such a model coupled with (i) dereferenceable Uniform Resource Identifiers (URI’s) for creating globally unique names, and (ii) standard languages such as RDFS, OWL, and SPARQL for creating ontologies as well as modeling and querying data, provides a very powerful framework for heterogeneous data integration.

While most clinical and research data is typically stored using relational databases (e.g., Oracle, MySQL) and queried using Structured Query Language (SQL), such technologies have several inherent limitations compared to RDF: (i) First, when database schemas are changed in a relational model, the whole repository, table structure, index keys etc. have to be reorganized—a task that can be quite complex and time-consuming. RDF, on the other hand, does not distinguish between schema (i.e., ontology classes and properties) and data (i.e., instances of the ontology classes) changes—both are merely addition or deletion of RDF triples, making such a model very nimble and flexible for updates. (ii) Second, RDF resources are identified by (globally) unique URI’s, thereby allowing anyone to add additional information about the resource. For example, via RDF links, it is possible to create references between two different RDF graphs, even in completely different namespaces, enabling much easier data linkage and integration. This is rather difficult to achieve in the classical relational database paradigm. (iii) Third, a relational data model lacks any inherent notion of a hierarchy. For instance, simply because a particular drug is an angiotensin receptor blocker (ARB), a typical SQL query engine (without any ad-hoc workarounds) cannot reason that it belongs to a class of anti-hypertensive drugs. Such queries are natively supported in RDFS and OWL. (iv) Finally, due to the lack of a formal temporal model for representing relational data, SQL provides minimal support for temporal queries natively. Such extensions are already in place for SPARQL [10].

In summary, linked data, and its enabling technologies such as RDF, provide a more robust, flexible, yet scalable model for integrating and querying data, thereby warranting investigation as to how such technologies can be applied in a clinical and translational research environment. However, while on one hand, such a huge integrated-network dataset provides exciting opportunities to execute expressive federated queries and integrating and analyzing information spanning genes, proteins, pathways, diseases, drugs, and adverse events, several questions remain unanswered about its applicability to high-throughput phenotyping of patient data in EHR systems. In the remainder of this paper, we provide a brief overview of RDF and SPARQL—the building blocks for linked data—and then propose our methods and present preliminary results in using Semantic Web technologies for mining DDI information from Mayo’s EHR systems.

### DrugBank and Drug-Drug Interaction Information

DrugBank [11] is a publicly available rich source of annotated drugs and drug target information. At the time of writing this manuscript in December 2012, DrugBank contained 6711 drug entries including 1447 FDA-approved small molecule drugs, 131 FDA-approved biotech (protein/peptide) drugs, 85 nutraceuticals and 5080 experimental drugs. Additionally, 4227 non-redundant protein (e.g., drug transporter or carrier) sequences are linked to these drug entries. The data is represented via a DrugCard where each entry comprising 150 data fields contains information on drug/chemical and drug target or protein data. Specifically, the data fields include information drug-action pathways, drug transporter data, drug metabolite data, pharmacogenomic data, adverse drug response data, ADMET data, pharmacokinetic data, computed property data and chemical classification data, and more recently drug-drug and drug-food interactions. Additionally, the DrugBank data is also available as RDF via the Bio2RDF [12] SPARQL endpoint. Our objective is to use the SPARQL endpoint to demonstrate how we can integrate publicly available data with institutional EHR data in a flexible manner. Since our use case for this work is investigate DDI information for Clopidogrel (Plavix), Warfarin (Coumadin) and several Protein Pump Inhibitors, we restrict our SPARQL query searches to these entities (see more details in Section 3.2). Table 1 below illustrates the DDI information extracted from DrugBank for these drugs.

## Materials and Methods

### Representing patient records as RDF graphs

**Figure 1:**
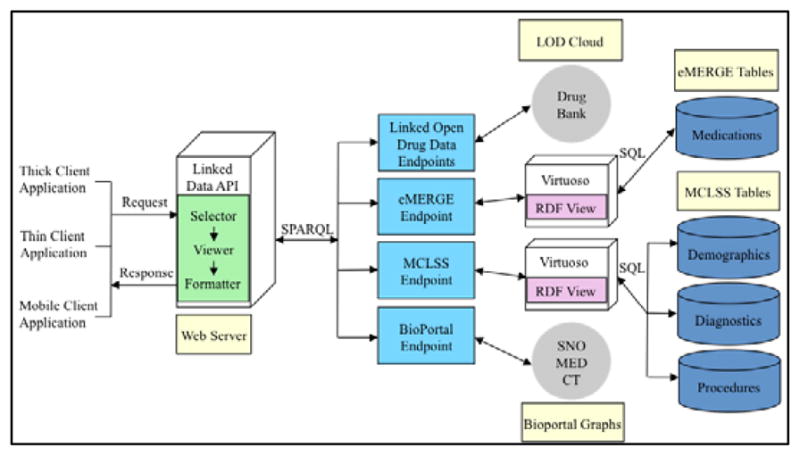
System architecture for representing patient EHR data as RDF and federated SPARQL querying

Figure 1 shows the proposed architecture for representing patient health records from MCLSS using RDF, linked data and related technologies. It comprises of two main components: (1) data access and storage, and (2) SPARQL-based querying interface. Here we provide a brief overview of these components; a detailed description of the architecture can be reviewed in our prior work [3].

#### Data Access and Storage

This component comprises the patient demographics, diagnoses, procedures, laboratory results, and free-text clinical and pathology notes generated during a clinical encounter. For accessing the clinical data, we query MCLSS via the DDQB programmatic interface as well as via direct JDBC and SQL queries. In particular, since our goal is to represent the data stored in the MCLSS database as RDF data, we use the open-source Virtuoso Universal Server [13] that acts as a mediator in the creation of materialized RDF graphs as well as provide a SPARQL endpoint for querying the graphs. In particular, a declarative language is used to describe the mappings between the relational schema and RDFS/OWL ontologies to create the RDF triples. This language generates a mapping file from table structures of the databases in MCLSS which can then be customized by replacing the auto-generated terms with concepts from standardized ontologies. In our case, we modified the custom ontology generated by Virtuoso for creating these mappings with terms and concepts from standardized SNOMED [14] ontology.

#### SPARQL endpoint

The RDF graphs created from MCLSS using the above approach were exposed via a SPARQL endpoint in the Virtuoso server. This allows software application clients to query the MCLSS RDF data using the SPARQL query language. Given that our overarching goal is to integrate the MCLSS and DrugBank RDF graphs, our objective is to execute federated queries across multiple SPARQL endpoints. We discuss the details of SPARQL-based federated querying in the next section.

### SPARQL-based federated querying for drug-drug interaction information and evidence

Figure 1 illustrates our goal to federate between two main data sources: MCLSS and DrugBank, where the former is a DB2 database containing patient clinical and demographic data, and the latter is a public drug data repository. Since our interest lies in querying for DDI pairs in DrugBank and determining such potential interactions using Mayo’s EMR prescription data, the first step is to simply extract the DDI knowledge for Cloplidogrel (Plavix) from DrugBank. This gives us a list of drug names—both generic and brand—as text, although without any association to standardized terminology codes, such as RxNorm [15] concept unique identifiers (RxCUIs). However, since MCLSS medication data is encoded using RxNorm codes, we have to first map the DDI pairs to appropriate RxCUIs before checking for potential DDI evidence within the patient prescription data. In its current form, one would have to execute a multiple SQL query across all these datasources to retrieve the appropriate resultset. Instead, by leveraging RDF and Semantic Web technologies, we demonstrate how this can be achieved using a single SPARQL query.

**Figure 2:**
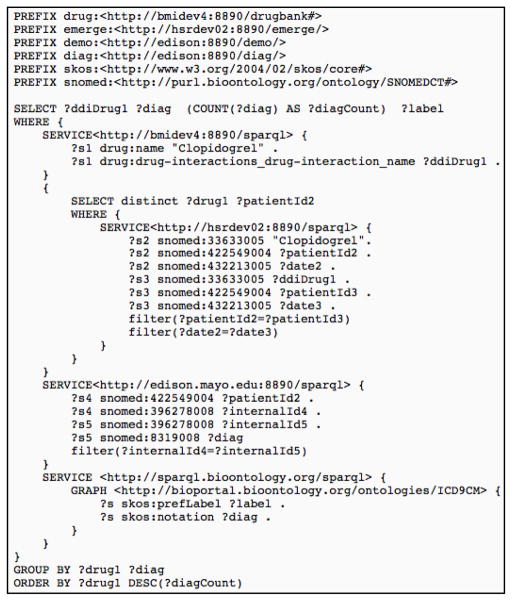
Federated SPARQL query for medication and diagnosis data with RxNorm codes

In particular, Figure 2 shows a snapshot of the federated SPARQL query that retrieves medication and diagnosis data for one of the DDI pairs: Clopidogrel and Warfarin. Specifically, the query illustrates how the SPARQL 1.1 SERVICE keyword is used to query and federate between two different SPARQL endpoints. In the first SERVICE stanza, the MCLSS SPARQL endpoint is being queried to provide the Mayo Clinic identification number for each patient along with their diagnosis such that there is evidence of two drugs—indicated via ?drug1 and ?drug2—in their prescription history. Both these drugs—in Figure 2—correspond to Clopidogrel and Warfarin whose corresponding RxCUIs are retrieved from a different SPARQL endpoint as indicated by the second SERVICE stanza. The complete SPARQL query retrieving data from the DrugBank SPARQL endpoint is accessible from our project site: http://informatics.mayo.edu.

## Results

We retrieved DDI information from the prescription medication data using the SPARQL endpoints for Clopidogrel, Warfarin and multiple Protein Pump Inhibitors on a cohort of 6758 patients that are part of Mayo’s eMERGE (Electronic Medical Records and Genomics) study [16]. This cohort primarily comprises of elderly patients who have been diagnosed with cardiovascular diseases; a detailed description of the cohort is presented elsewhere [16]. Table 2 shows the demographics on a subset of the eMERGE cohort highlighting only those patients who were prescribed both Clopidogrel and the PDDI medication.

Out of the 11 potential DDIs for Clopidogrel listed in Table 1, only six were observed in the prescription medication dataset (. While the gender distribution was not biased, the potential DDI evidences were observed primarily on an older population. This would be consistent with the fact that cardiovascular diseases are predominantly observed in elderly, and hence higher incidence of Clopidogrel prescriptions. Table 3 provides another dimension of these findings in the context of patient diagnoses, which further illustrates the prevalence of cardiovascular diseases and its risk factors in this cohort.

Of particular interest, are the DDI pairs for Protein Pump Inhibitors (DDI_3, DDI_5, DDI_7, DDI_8) and Warfarin (DDI_11). In a large outcomes study conducted by Medco Health Solutions in 2009, it was reported that concomitant therapy for Clopidogrel with Protein Pump Inhibitors, including Pantoprazole (brand name: Protonix), should be avoided since it can increase the risk of major cardiovascular problems by 50 percent and having a heart attack by 74 percent [17]. Consistent with such a recommendation, our data indicates a significant drop in concomitant therapy for Clopidogrel and Protein Pump Inhibitors. Similarly, several research studies [18] and the American College of Cardiology and American Heart Association [19] recommend combining antiplatelets and anticoagulants with a low-dose of Aspirin at a narrow international normalized ratio (INR) range of 2.0–3.0, although concomitant administration of Aspirin, Clopidogrel and Warfarin is considered a matter of clinical judgment. Specifically, additional factors such as duration of the therapy and safety (e.g., risk of bleeding) are always unequivocally taken into consideration. Our data suggests a similar pattern indicating lack of consensus on practice guidelines and recommendations.

## Discussion

### Summary

Research in clinical and translational science demands effective and efficient methods for accessing, integrating, interpreting and analyzing data from multiple, distributed, and often heterogeneous data sources in a unified way. Traditionally, such a process of data collection and analysis is done manually by investigators and researchers, which is not only time consuming and cumbersome, but in many cases, error prone. The emerging Semantic Web tools and technologies allow exposing different modalities of data, including clinic, research, and scientific, as structured RDF that can be queried uniformly via SPARQL. Not only does this provide the capabilities for interlinking and federated querying of diverse Web-based resources, but also enables fusion of private and public data in very powerful ways.

The overarching goal of this study is to precisely explore federated data integration and querying using public data sources from the Linked Open Data cloud, and private, identifiable patient data from Mayo Clinic’s EHR systems. Using open-source tooling and software, we developed a proof-of-concept system that allows representing patient data stored in Mayo’s enterprise warehouse system as RDF, and exposing it via a SPARQL endpoint for accessing and querying. Our use case for federated querying of DDI information from DrugBank further demonstrated the applicability of such a system and the benefits of interlinking and querying multiple, heterogeneous Web data sources that are publicly available, with private (and institution-specific) patient information. We hypothesize that further development of such a system can immensely facilitate, and potentially accelerate scientific findings in clinical and translational research.

### Limitations

The proof-of-concept system developed in this study has several limitations. First, while we demonstrated the applicability of the system via sample use case queries, a more robust and rigorous evaluation along several dimensions (e.g., performance, query response, precision and recall of query results etc.) is required before it can be deployed within an enterprise environment. Note that since our use cases are based on federated querying of several public SPARQL endpoints, the system performance and query responses are dependent on the behavior of the endpoints. Nevertheless, we plan to perform a thorough system evaluation after the integration of additional MCLSS sources (e.g., laboratory, clinical and pathology reports) that contain large amounts of patient data. Second, the use-case queries were executed on a small cohort of approximately 7000 patients, and only drug prescription data was available. It remains to be seen if the preliminary findings for the DDI pairs can be replicated in a larger cohort, and more importantly, using drug administration data. Finally, while one of the Linked Data principles is to make data publicly available and accessible, due to privacy and HIPPA constraints of identifiable patient data, the MCLSS RDF views remain private. Consequently, only appropriate personnel within Mayo’s firewall approved by Mayo’s Institutional Review Board participating in this study can access our application.

### Future Work

In addition to addressing the limitations aforementioned, there are several activities that we plan to pursue in the future. Firstly, in this study, we studied only a handful of DDI pairs. Our immediate goal is to expand the DDI pair list that are of clinical significance and consider both drug prescription and administration data. Secondly, our experience in discussing and demoing the proof-of-concept to clinicians made it amply clear that we should focus on developing visual and interactive interfaces for forming the SPARQL queries. To this end, we plan to explore visual SPARQL editing tools, such as SPARQLMotion [20]. Finally, as depicted in Figure 1, we plan to use the Linked Data API for creating our service layer to provide application developers a friendlier access to the data, for example, using JSON.

## Figures and Tables

**Table 1:** Potential drug-drug interaction information for Clopidogrel

Drug Name	Interaction Type
Bortezomib	May decrease serum concentrations of the active metabolite(s) of Clopidogrel
Drotrecogin Alfa	May enhance the adverse or toxic effect of Drotrecogin Alfa during concomitant therapy
Esomeprazole	May decrease serum concentrations of the active metabolite(s) of Clopidogrel
Ginkgo Biloba	May increase risk of bleeding during concomitant therapy with Clopidogrel
Lansoprazole	May decrease serum concentrations of the active metabolite(s) of Clopidogrel
Omeprazole	May decrease serum concentrations of the active metabolite(s) of Clopidogrel
Pantoprazole	May decrease serum concentrations of the active metabolite(s) of Clopidogrel
Rabeprazole	May decrease serum concentrations of the active metabolite(s) of Clopidogrel
Rivaroxban	Clopidogrel may enhance the anticoagulant effect of Rivaroxaban
Treprostinil	May increase risk of bleeding during concomitant therapy with Clopidogrel
Warfarin	May increase risk of bleeding during concomitant therapy with Clopidogrel

**Table 2:** Demographics of the eMERGE cohort (N=6758) for potential Clopidogrel DDIs

DDI Pair: Clopidogrel and	DDI Pair Unique ID	Gender: # Males	Gender: # Females	Age: 18–30	Age: 31–50	Age: ≥ 51
Esomeprazole	DDI_3	36	19	0	1	54
Ginkgo Biloba	DDI_4	2	1	0	0	3
Lansoprazole	DDI_5	22	21	0	2	41
Pantoprazole	DDI_7	77	46	0	3	120
Rabeprazole	DDI_8	12	9	0	0	21
Warfarin	DDI_11	132	62	0	8	186

**Table 3:** Top 5 ICD-9-CM based diagnosis for patients prescribed a Clopidogrel DDI

DDI Pair Unique ID	Top 5 observed ICD-9-CM diagnosis
DDI_3	Unspecified essential hypertension
	Other and unspecified hyperlipidemia
	Other forms of chronic ischemic heart disease
	Coronary atherosclerosis of native coronary artery
	Screening for malignant neoplasms of prostate
DDI_4	Other and unspecified hyperlipidemia
	Unspecified hereditary and idiopathic peripheral neuropathy
	Unspecified hearing loss
	Residual hemorrhoidal skin tags
	Mastodynia
DDI_5	Unspecified essential hypertension
	Other forms of chronic ischemic heart disease
	Other and unspecified hyperlipidemia
	Coronary atherosclerosis of native coronary artery
	Atherosclerosis of native arteries of the extremities with intermittent claudication
DDI_7	Unspecified essential hypertension
	Other and unspecified hyperlipidemia
	Other forms of chronic ischemic heart disease
	Coronary atherosclerosis of native coronary artery
	Diabetes mellitus
DDI_8	Other and unspecified hyperlipidemia
	Unspecified essential hypertension
	Diabetes mellitus
	Other specified pre-operative examination
	Screening for malignant neoplasms of prostate
DDI_11	Unspecified essential hypertension
	Other and unspecified hyperlipidemia
	Other forms of chronic ischemic heart disease
	Coronary atherosclerosis of native coronary artery
	Diabetes Mellitus

## References

[1] Bizer C, Heath T, Berners-Lee T (2009). Linked Data - The Story So Far. International Journal on Semantic Web and Information Systems.

[2] Resource Description Framework (RDF).

[3] Pathak J, Kiefer R, Chute C (2012). Applying Linked Data Principles to Represent Patient’s Electronic Health Records at Mayo Clinic: A Case Report.

[4] Pathak J, Kiefer R, Chute CG (2012). Using Semantic Web Technologies for Cohort Identification from Electronic Health Records to Conduct Genomic Studies.

[5] Greenberg MRM (2011). CLinical decision support and malpractice risk. JAMA: The Journal of the American Medical Association.

[6] Ramirez AH (2012). Predicting warfarin dosage in European–Americans and African–Americans using DNA samples linked to an electronic health record. Pharmacogenomics.

[7] Delaney JT (2012). Predicting Clopidogrel Response Using DNA Samples Linked to an Electronic Health Record. Clin Pharmacol Ther.

[8] Kurland L, Molgaard C (1981). The Patient Record in Epidemiology. Scientific American.

[9] Weiss T (2002). IBM, Mayo Clinic to develop database for clinical trials, research.

[10] Tappolet J, Bernstein A Applied Temporal RDF: Efficient Temporal Querying of RDF Data with SPARQL.

[11] Knox C (2011). DrugBank 3.0: a comprehensive resource for ‘Omics’ research on drugs. Nucleic Acids Research.

[12] Belleau F (2008). Bio2RDF: Towards a mashup to build bioinformatics knowledge systems. Journal of Biomedical Informatics.

[13] Virtuoso Universal Server http://virtuoso.openlinksw.com/.

[14] SNOMED-CT: Systematized Nomeclature of Medicine-Clinical Terms.

[15] Nelson SJ (2011). Normalized names for clinical drugs: RxNorm at 6 years. Journal of the American Medical Informatics Association.

[16] Kullo I (2010). Leveraging Informatics for Genetic Studies: Use of the Electronic Medical Record to Enable a Genome-Wide Association Study of Peripheral Arterial Disease. JAMIA.

[17] (2009). Medco study: PPIs reduce clopidogrel efficacy post-stenting.

[18] Hermosillo AJ, Spinler SA (2008). Aspirin, Clopidogrel, and Warfarin: Is the Combination Appropriate and Effective or Inappropriate and Too Dangerous?. The Annals of Pharmacotherapy.

[19] Anderson JL (2007). ACC/AHA 2007 Guidelines for the Management of Patients With Unstable Angina/Non–ST-Elevation Myocardial Infarction. Circulation.

[20] Waldman S (2011). TopQuadrant: SPARQLMotion Visual Scripting Language.

